# Preclinical Evaluation of Engineered Oncolytic Herpes Simplex Virus for the Treatment of Neuroblastoma

**DOI:** 10.1371/journal.pone.0077753

**Published:** 2013-10-10

**Authors:** Lauren A. Gillory, Michael L. Megison, Jerry E. Stewart, Elizabeth Mroczek-Musulman, Hugh C. Nabers, Alicia M. Waters, Virginia Kelly, Jennifer M. Coleman, James M. Markert, G. Yancey Gillespie, Gregory K. Friedman, Elizabeth A. Beierle

**Affiliations:** 1 Department of Surgery, Division of Pediatric Surgery, University of Alabama, Birmingham, Birmingham, Alabama, United States of America; 2 Department of Pathology, Children’s of Alabama, Birmingham, Alabama, United States of America; 3 Department of Pediatrics, Division of Hematology/Oncology, University of Alabama, Birmingham, Birmingham, Alabama, United States of America; 4 Department of Surgery, Division of Neurosurgery, University of Alabama, Birmingham, Birmingham, Alabama, United States of America; University of Chicago, United States of America

## Abstract

Despite intensive research efforts and therapeutic advances over the last few decades, the pediatric neural crest tumor, neuroblastoma, continues to be responsible for over 15% of pediatric cancer deaths. Novel therapeutic options are needed for this tumor. Recently, investigators have shown that mice with syngeneic murine gliomas treated with an engineered, neuroattenuated oncolytic herpes simplex virus-1 (oHSV), M002, had a significant increase in survival. M002 has deletions in both copies of the γ_*1*_34.5 gene, enabling replication in tumor cells but precluding infection of normal neural cells. We hypothesized that M002 would also be effective in the neural crest tumor, neuroblastoma. We showed that M002 infected, replicated, and decreased survival in neuroblastoma cell lines. In addition, we showed that in murine xenografts, treatment with M002 significantly decreased tumor growth, and that this effect was augmented with the addition of ionizing radiation. Importantly, survival could be increased by subsequent doses of radiation without re-dosing of the virus. Finally, these studies showed that the primary entry protein for oHSV, CD111 was expressed by numerous neuroblastoma cell lines and was also present in human neuroblastoma specimens. We concluded that M002 effectively targeted neuroblastoma and that this oHSV may have potential for use in children with unresponsive or relapsed neuroblastoma.

## Introduction

Neuroblastoma, a tumor of neural crest origin, is the most common extra-cranial solid tumor of childhood, constituting 8-10% of all pediatric cancers [1]. Advances in therapy over the last several decades have improved the outcome for some children with this tumor, but there has been little change in the prognosis for advanced, metastatic and recurrent disease [2]. For these high risk tumors, survival rates remain less than 50% and novel therapies are needed to improve the outcomes for these children.

 Therapies with oncolytic viruses are currently being explored for other pediatric tumors and have shown some promise in the treatment of neuroblastoma [3]. Treatment of a syngeneic mouse model of neuroblastoma with an attenuated poliovirus resulted in a significant decrease in tumor growth, and subsequent challenge of cured mice with tumor cells did not result in tumor growth [4]. Similarly, the intratumoral injection of oncolytic vaccinia virus in a syngeneic murine neuroblastoma model resulted in decreased primary tumor growth [5]. An oncolytic adenovirus was used in a human neuroblastoma patient in Finland. These investigators reported the intratumoral injection of the virus in a child with refractory neuroblastoma, leading to a significant decrease in tumor volume with the child surviving 14 months after therapy [6]. Oncolytic herpes simplex virus (oHSV) mutants have also shown effectiveness in both murine and human neuroblastoma models [7-10]. 

 Parker et al. described an engineered oncolytic herpes simplex virus (M002) expressing both subunits of biologically active murine interleukin (IL)-12 in physiological concentrations [11]. M002 is a conditionally replication-competent virus with mutations in both copies of the γ_*1*_34.5 gene, located within the inverted repeats of the unique long segment. Herpes simplex virus-1 (HSV-1) is capable of infecting many different types of cells, but the natural latency of HSV occurs in neurons. Therefore, M002 preferentially localizes to neural tissue, such as the neural crest cells of neuroblastoma. Survival of syngeneic animals injected intracranially with Neuro-2a cells was significantly lengthened after intratumoral injection with M002, when compared to saline or injection of R3659, the parent HSV that lacked IL-12 [11]. With these data, we hypothesized that M002 would be cytotoxic to human neuroblastoma cells and decrease tumor growth in murine models of neuroblastoma. 

## Materials and Methods

### Cells and cell culture

All cell lines were maintained in culture under standard conditions at 37 °C and 5% CO_2_. Neuroblastoma cells were cultured as previously described [12]. Briefly, SK-N-AS (CRL-2137, American Type Culture Collection, ATCC, Manassas, VA) human neuroblastoma cells were maintained in Dulbecco’s modified Eagle’s medium containing 10% fetal bovine serum, 4 mM L-glutamine, 1 µM non-essential amino acids and 1 µg/mL penicillin/streptomycin. SK-N-BE(2) (CRL-2271, ATCC) and SH-SY5Y (CRL-2266, ATCC) human neuroblastoma cells were maintained in a 1:1 mixture of minimum Eagle’s medium and Ham’s F-12 medium with 10% fetal bovine serum, 2 mM L-glutamine, 1 µM non-essential amino acids and 1 µg/mL penicillin / streptomycin. SK-N-SH (HTB-11, ATCC) neuroblastoma cell line was maintained in modified Eagle’s medium containing 10% fetal bovine serum, 2 mM L-glutamine and 1 µg/mL penicillin / streptomycin. The SH-EP (MYCN-) and the isogenic WAC2 (MYCN+) human neuroblastoma cell lines were generously provided by Dr. M. Schwab (Deutsches Krebsforschungszentrum, Heidelberg, Germany), and have been described in detail previously [13]. These two cell lines were maintained in RPMI 1640 medium supplemented with 10% fetal bovine serum and 1 μg/mL penicillin / streptomycin. Neuro-2a murine neuroblastoma cells were obtained from our collaborator (G. Y. G.) and were maintained in culture with a 50:50 mixture of Dulbecco’s modified Eagle medium and Ham’s F-12 medium supplemented with 7% fetal bovine serum, 2 mM L-glutamine, 1 µM non-essential amino acids and 1 µg/mL penicillin / streptomycin. Vero cells were obtained from ATCC (CCL-81) and maintained using modified Eagle’s medium containing 7% fetal bovine serum, 2 µM L-glutamine and 1 µg/mL penicillin / streptomycin.

### Virus

A genetically-engineered, oncolytic herpes simplex virus, M002, has been previously described [11]. Briefly, R3659 was the parent virus for M002 with the thymidine kinase gene inserted into deleted regions of both γ_*1*_34.5 loci and a deletion in the native thymidine kinase locus. M002 is a conditionally replication-competent mutant herpes simplex virus expressing both subunits of murine IL-12 (mIL-12) under the transcriptional control of the murine Early-growth response-1 promoter (Egr-1); two copies of the entire construct are present, with a single copy inserted into each of the γ_*1*_34.5 loci; the native thymidine kinase gene is restored. To titer the M002 and R3659 virus, Vero cells were plated in 24 well plates at 1.5 x 10^5^ cells per well and allowed 24 hours to attach and form a confluent monolayer. Ten-fold dilutions of stock virus in infection medium (1% FBS in DMEM/F12) were applied to the Vero cells for 2 hours, then the inoculum was removed and the plates washed with media. After an additional 48 hours of incubation, May-Grunwald stain in methanol was applied for 20 minutes plates were washed and allowed to dry overnight. Plaques were counted and the titer calculated and reported as plaque-forming units per milliliter (pfu/mL). 

### Virus cytotoxicity assays

Cell viability 72 hours after virus treatment *in vitro* was measured using an alamarBlue® assay. Briefly, cells were plated (1.5 × 10^3^ cells/100 μL well) in 96-well culture plates and after 24 hours were treated with 100 μL of saline or a graded series of dilutions of M002. After 66-68 hours of culture, 10 μL of sterile alamarBlue® dye (Invitrogen Life Technologies, Grand Island, NY) was added to each well. After 4-6 hours, the absorbance at 542 and 595 nm was measured using a kinetic microplate reader (BioTek Gen5, BioTek Instruments, Winooski, VT). Virus cytotoxicity at each dilution was measured by the reduction in the color change compared with that seen in the saline treatment group (100%) viability. These values were plotted to yield an estimate of the numbers of plaque-forming units (PFU) of M002 needed to kill 50% of the cells by 72 hrs (LD_50_/PFU).

### Viral replication

For multi-step viral recovery experiments, SK-N-AS and SK-N-BE(2) cells were grown to confluence in 6-well plates and then infected with M002 at a multiplicity of infection (MOI) of 0.1 PFU/cell. The media from the cells were harvested at 6, 24, 48, and 72 hours post-infection. The titers of progeny virions in the supernate were determined on monolayers of Vero cells, and the average number of PFU/mL was calculated from quadruplicate wells. 

 Single step viral recovery experiments were performed as previously described [14]. In brief, SK-N-AS and SK-N-BE(2) cells were plated and allowed to attach for 24 hours. The cells were infected with M002 at a MOI of 10 PFU/cell for 2 hours. After 12 and 24 hours, the cells were harvested by adding equal volume of sterile milk and freezing at -80 °C. Plates were thawed at 37 °C and underwent two more cycles of freeze / thaw. Cells and supernates were collected, milk stocks sonicated for 30 seconds, and the titers of progeny virions were determined on monolayers of Vero cells. The average number of PFU/mL was calculated from quadruplicate wells. 

### Murine IL-12 ELISA

Production of murine IL-12 by the recombinant M002 virus was quantified using a total murine IL-12 ELISA kit (Thermoscientific, Rockford, IL). Ninety-six well plates were seeded with 1.5 × 10^4^ cells per well for 24 hours and then treated with media alone or M002. After 48 hours of incubation at 37 °C, the supernates were collected and analyzed by ELISA, according to the manufacturer’s protocol.

### Ethics Statement

All animal experiments were performed after obtaining protocol approval by the UAB Animal Care and Use Committee (120409363), and in compliance with the recommendations in the Guide for the Care and Use of Laboratory Animals of the National Institutes of Health. The human subject samples were obtained after protocol approval by the UAB institutional review board (IRB X111123007) under waiver of informed consent.

### Tumor growth in vivo

Six week old female athymic nude mice were purchased from Harlan Laboratories, Inc. (Chicago, IL). The mice were maintained in the SPF animal facility with standard 12 hour light / dark cycles and allowed chow and water *ad libitum*. At the completion of the experiments, euthanasia was accomplished according to American Association for Laboratory Animal Science (AALAS) guidelines utilizing compressed CO_2_ gas in cylinders in their home cage followed by cervical dislocation. Human neuroblastoma cells, SK-N-AS (2.5 ×10^6^ cells) and SK-N-BE(2) (2.5 ×10^6^ cells) in Matrigel™ (BD Biosciences, San Jose, CA) were injected subcutaneously into the right flank. Once tumors had reached approximately 300 mm^3^, animals received an intra-tumoral injection of vehicle (PBS + 10% glycerol, 50μL) or M002 virus (50μL). For the first experiment, the animals received intratumoral injections of either vehicle alone (n=5) or M002 [1 × 10^7^ PFU/50μL (n=5)]. These concentrations were based upon previous investigations with the virus [11]. Tumors were measured twice weekly with a caliper and tumor volume in mm^3^ was calculated using a standard formula [(width^2^ × length)/2], where width was the smaller diameter. Once tumors reached the size predetermined by protocol standards, the animals were euthanized and the tumors were harvested and processed for study. For the second study, after SK-N-AS and SK-N-BE(2) flank xenografts were established, the animals were treated with intratumoral injection of either vehicle or M002 at low dose (1 × 10^4^ PFU/50μL). Since previous studies demonstrated that irradiation increased the efficacy of the virotherapy [15,16], within 24 hours of vehicle or M002 injection, half of each group was irradiated with 3 gray (Gy) directed at the flank tumor. There were 5 animals per group. Tumors were measured twice weekly with a caliper, tumor volume was calculated, and once tumors reached the size predetermined by protocol, the animals were euthanized and the tumors were harvested and processed for study. 

 For the third xenograft study, we examined whether repeated exposure to ionizing radiation would be as effective as repeated dosing of M002. Once SK-N-AS and SK-N-BE(2) flank xenografts were established (300 mm^3^), the animals were treated with intra-tumoral injection of M002 [1 × 10^4^ PFU/50μL (N=15)] followed by low dose (3 Gy) ionizing irradiation directed at the flank tumors. The animals were separated into 3 treatment groups (n=5 per group). One group had no further treatment (M002 + XRT). The second group received a repeat low dose of XRT (3 Gy) directed to the tumor (M002 + XRT × 2), and the third group received a second dose of intra-tumoral M002 (1 × 10^4^ PFU / 50μL) with a low dose XRT (3 Gy) [(M002 + XRT) × 2]. Tumor volumes were measured twice per week, and once tumors reached the size predetermined by protocol, the animals were euthanized. Data reported as fold change in tumor volume ± standard error. 

 In the final xenograft study, we examined whether repeated exposure to ionizing radiation would lead to continued tumor cell killing. Once SK-N-AS and SK-N-BE(2) flank xenografts were established, the animals were treated with intra-tumoral injection of vehicle or M002 (1 × 10^4^ PFU/50μL). Within 24 hours of injection, the animals were irradiated with 3 Gy directed at the flank tumors and separated into 5 treatment groups (n=5 per group). At one week intervals, the animals were given a repeat low dose of radiation (3Gy) to the tumor. Tumor volumes were measured twice weekly, and once tumors reached the size predetermined by protocol, the animals were euthanized. 

 To determine the affects of murine IL-12 upon tumor growth, an immunocompetent syngeneic murine model of neuroblastoma was utilized. Six week old female AJ immunocompetent mice were purchased from Fredrick Cancer Research (Hartford, CT). The mice were maintained in the SPF animal facility with standard 12 hour light / dark cycles and allowed chow and water *ad libitum*. At the completion of the experiments, euthanasia was accomplished according to American Association for Laboratory Animal Science (AALAS) guidelines utilizing compressed CO_2_ gas in cylinders in their home cage followed by cervical dislocation. Neuro-2a murine neuroblastoma cells (5 × 10^5^ cells) were injected into the right flank of syngeneic immunocompetent AJ mice. Once tumors reached approximately 100 mm^3^, animals received an intra-tumoral injection of R3659 (parent, n=8) or M002 (murine IL-12 expressing, n=7) virus in equal concentrations (1 × 10^7^ PFU/50μL). Tumor volumes were measured twice weekly, and once tumors reached the parameters predetermined by IACUC protocol, the animals were euthanized. 

### Flow cytometry analysis

The CD111 and CD112 expression in neuroblastoma cell lines was measured with flow cytometry. For staining, neuroblastoma cells were harvested and centrifuged at 900 rpm for 4 minutes. The cell pellet was pipetted with autoMACS® running buffer (130-091-221, Miltenyi Biotec, Bergisch Gladbach, Germany) to obtain a single cell suspension. Cells were blocked with 20µL FcR Blocker (120-000-442, Miltenyi Biotec) and cells with blocker alone served as negative controls. Phycoerythrin (PE) conjugated anti-human CD111 antibody (340403, Biolegend, San Diego, CA) or PE anti-human CD112 antibody (337410, Biolegend) was added and cells incubated at 4 °C in the dark for 20 minutes. Cells were again centrifuged and a single cell suspension was obtained with autoMACS® buffer (Miltenyi Biotec). Cells were analyzed with fluorescence-activated cell sorting (FACS) using a BD LSR II Flow Cytometer (BD Biosciences, San Jose, CA). Data were analyzed with FlowJo v10.0.6 (Tree Star Inc., Ashland, OR).

 The presence of natural killer (NK) cells was detected in the neuroblastoma xenograft tumors using flow cytometry. The tumors were harvested, mechanically disassociated, and passed through a 100 μm strainer. The specimens were centrifuged at 900 rpm for 4 minutes. The cell pellet was pipetted with autoMACS® running buffer (Miltenyi Biotec) to obtain a single cell suspension. Specimens were blocked with 20µl FcR Blocker (Miltenyi Biotec) and those with blocker alone served as negative controls. Additional controls were included with each experiment consisting of normal murine splenocytes and tumor cells alone [SK-N-AS or SK-N-BE(2)]. FITC conjugated anti-mouse Ly49D antibody (138304, clone 4E5, Biolegend) was added and the specimens incubated at 4 °C in the dark for 20 minutes. Specimens were then washed with autoMACS® buffer (Miltenyi Biotec) and again centrifuged. Finally, specimens were fixed in 4% paraformaldehyde and analyzed with fluorescence-activated cell sorting (FACS) using a BD LSR II Flow Cytometer (BD Biosciences). Data were analyzed with FlowJo v10.0.6 (Tree Star Inc.). Of note, other NK antibodies were also tested for FACS analysis but were found to either not recognize nude mouse NK cells (eBioscience anti-mouse CD335, clone 29A1.4, 11-3351) or cross-react with glycolipids known to be present on the tumor cells themselves (Biolegend anti-mouse CD49b, clone DX5, 108902).

### Antibodies

Antibodies used for Western blotting were as follows: rabbit polyclonal anti-phospho Stat1 (Y701, 9171S) and anti-Stat1 (9172S) were obtained from Cell Signaling (Cell Signaling Technology, Inc., Danvers, MA); mouse monoclonal anti-phospho p38 MAPK (Thr180/Tyr182, 9216S) was from Cell Signaling and rabbit polyclonal anti-p38 (H-147, sc-7149) was obtained from Santa Cruz (Santa Cruz Biotechnology Inc., Santa Cruz, CA); and GAPDH antibody was from Fitzgerald (10R-1178, Fitzgerald Industries International, Acton, MA). 

### Western blotting

Western blots were performed as previously described [17]. Briefly, cells were lysed on ice for 30 min in a buffer containing 50mM Tris-HCL, (pH 7.5), 150 mM NaCl, 1% Triton-X, 0.5% NaDOC, 0.1% SDS, 5mM EDTA, 50mM NaF, 1 mM NaVO3, 10% glycerol, and protease inhibitors: 10 μg/mL leupeptin, 10 μg/mL PMSF and 1 μg/mL aprotinin. The lysates were cleared by centrifugation at 14 000 rpm for 30 minutes at 4 °C. Protein concentrations were determined using a Bio-Rad kit (Bio-Rad, Hercules, CA) and proteins were separated by electrophoresis on SDS-PAGE gels. Antibodies were used according to manufacturers’ recommended conditions. Molecular weight markers (Precision Plus Protein Kaleidoscope Standards, Bio-Rad) were used to confirm the expected size of the target proteins. Immunoblots were developed with chemiluminescence (Amersham ECL Western blotting detection reagents) (GE Healthcare Biosciences, Pittsburgh, PA). Blots were stripped with stripping solution (Bio-Rad) at 37 °C for 15 minutes, rinsed and then reprobed with selected antibodies. Immunoblotting with antibody to GAPDH provided an internal control for equal protein loading. 

### Immunohistochemistry

Formalin-fixed, paraffin-embedded tumor blocks for the human specimens or murine xenografts were cut in 8 micrometer sections. The slides were baked for 1 hour at 70 °C, deparaffinized, rehydrated, and steamed. The sections were then quenched with 3% hydrogen peroxide and blocked with PBS-blocking buffer. The primary rabbit polyclonal antibodies, anti-CD111 antibody (1:200, ab66985, Abcam, Cambridge, MA) or anti-Herpes Simplex Virus Type I antibody (1:250, PU084-UP, BioGenex, Fremont, CA) were added and incubated overnight at 4 °C. After washing with PBS, the Superpicture anti-rabbit HRP secondary antibody (Life Technologies, Inc., Grand Island, NY) was added 1:250 dilution for 1 hour at 22 °C. The staining reaction was developed with VECTASTAIN Elite ABC kit (PK-6100, Vector Laboratories, Burlingame, CA), TSA™ (biotin tyramide reagent, 1:400, PerkinElmer, Inc., Waltham, MA) and DAB (Metal Enhanced DAB Substrate, Thermo Fisher Scientific, Rockford, IL). Slides were counterstained with hematoxylin. Negative controls (rabbit IgG, 1 µg/mL (Millipore, EMD Millipore, Billerica, MA) were included with each experiment. For hematoxylin and eosin staining, slides were cut and baked as described above and standard H&E staining methods were utilized. The human subject samples were obtained after protocol approval by the UAB institutional review board (IRB X111123007) under waiver of informed consent.

### Immunohistochemistry Scoring

For CD111 staining, a single board-certified pathologist (E.M.M.), blinded to the specimens, reviewed each human tissue section for CD111 and assigned a stain score. The slides were examined and the staining evaluated by measuring the intensity of stain and assigned a score (0, none; 1, weak; 2, moderate; 3, strong; 4, extremely strong). Staining for HSV-1 in xenograft tumors was quantified by ImageJ software (http://rsb.info.hin.gov/ij/). Positive HSV-1 staining was reported as percent positive staining cells per high power field after counting 10 random fields of view per specimen. 

### Data analysis

Experiments were repeated at least in triplicate, and data reported as mean ± standard error of the mean (SEM). Densitometry of immunoblots was performed utilizing Scion Image Program (http://www.nist.gov/lispix/imlab/prelim/dnld.html). Bands were normalized to those of GAPDH then compared to each other where appropriate. An ANOVA or Student’s t-test was used as appropriate to compare data between groups and log-rank test was used to determine survival significance. Statistical analyses were completed using SigmaPlot™ 12 software (SyStat Software, Inc., San Jose, CA) with statistical significance determined at the p ≤ 0.05 level.

## Results

### *In vitro* sensitivity of neuroblastoma cells to treatment with M002

**Figure 1:**
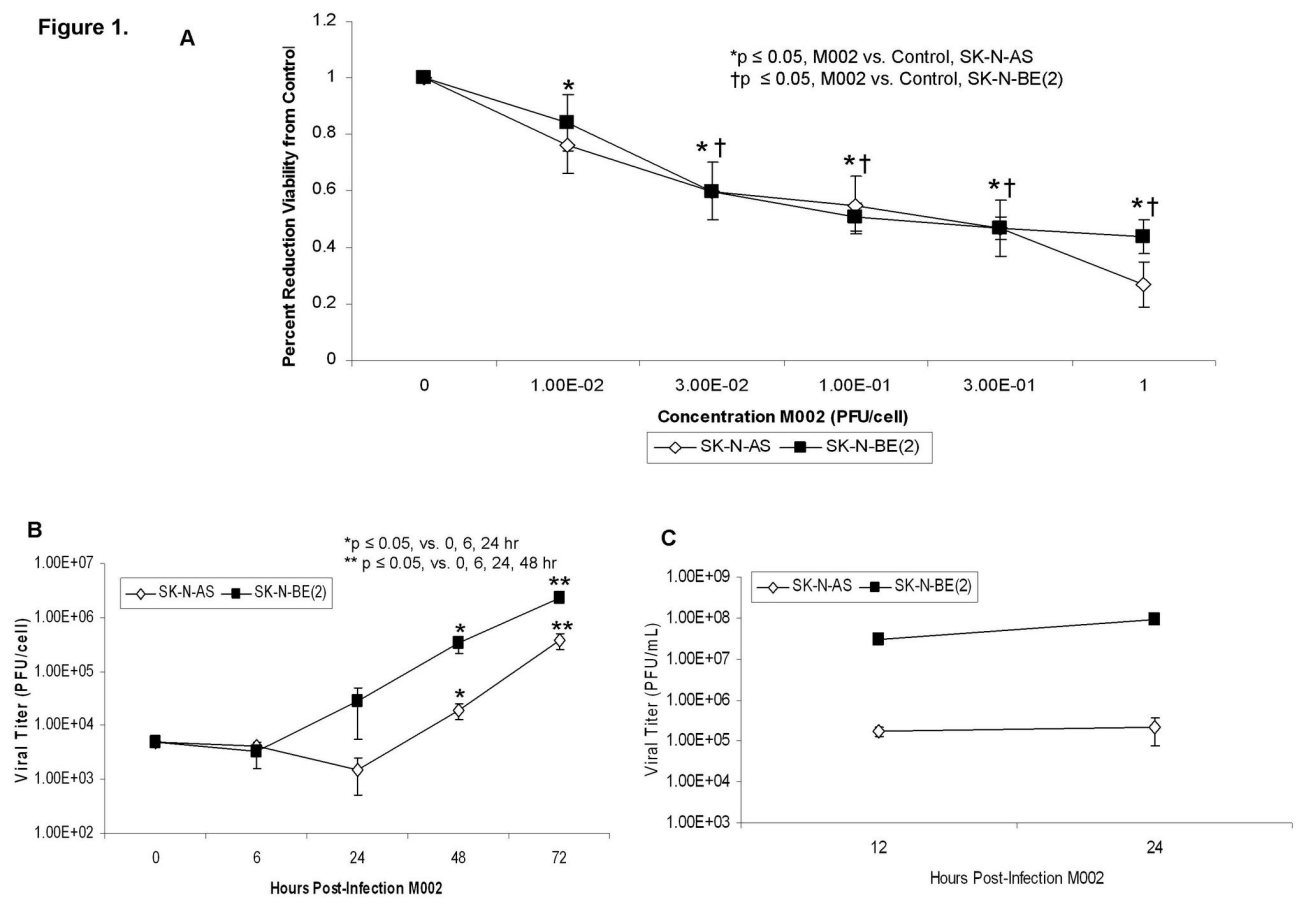
M002 infection and cell survival in neuroblastoma cell lines. **A** SK-N-AS and SK-N-BE(2) cell lines were treated with M002 at increasing MOI. After 72 hr of treatment, cell viability was measured with alamarBlue™ assays. Data reported as mean ± standard error of the mean. Both cell lines had a significant decrease in viability following M002 treatment. B Multi-step replication of M002. Monolayers of SK-N-AS or SK-N-BE(2) cells were infected with M002 at a MOI of 0.1 PFU/cell, and at 6, 24, 48, 72 hours post-infection, supernates were collected and virus titers determined on Vero cell monolayers. Mean virion yields were determined in four replicates at each time point and standard error of the mean was determined. M002 replicated nearly 2 logs higher than control at 72 hours post-infection. C Single-step *in* *vitro* replication of M002. Monolayers of SK-N-AS or SK-N-BE(2) cells were infected with M002 at a MOI of 10 PFU/cell. Replicate cultures were harvested at 12 and 24 hours post-infection and virus titers determined on Vero cell monolayers. Mean virion yields were determined in four replicates at each time point and standard error of the mean was determined.

We chose two neuroblastoma cell lines, SK-N-AS and SK-N-BE(2) as both of these cell lines form subcutaneous tumors in nude mice [17] and they have differing status of *MYCN* amplification, the most important negative prognostic factor for neuroblastoma [13]. SK-N-AS cells are *MYCN* non-amplified [18] and SK-N-BE(2) cells are *MYCN* amplified [19]. The neuroblastoma cell lines were treated with M002 at increasing concentrations [plaque forming units/cell (PFU/cell)] and cell viability was measured with alamarBlue® assays after 72 hours of treatment. Both cell lines had a significant decrease in viability following M002 treatment (Figure 1. **A**). The lethal dose of virus that resulted in 50% cell death (LD_50_) for the SK-N-AS cells was 0.37 ± 0.1 PFU/cell and for the SK-N-BE(2) cells was 0.17 ± 0.05 PFU/cell. Additional neuroblastoma cell lines tested were also highly sensitive to killing by M002 with a LD_50_ ranging from 0.09 to 1.06 PFU/cell (Table 1). 

**Table 1:** Neuroblastoma cell lines M002 LD_50_ dose and CD111^+^ staining.

**Neuroblastoma Cell Line**	***MYCN***	**M002 LD_50_ (PFU/cell)**	**CD111^+^**
		**(Mean ± SEM)**	**(Percentage ± SEM)**
SK-N-AS	Non-amplified	0.37 ± 0.1	9.8 ± 1.7
SK-N-BE(2)	Amplified	0.17 ± 0.05	93.2 ± 2.7
SH-SY5Y	Non-amplified	0.09 ± 0.01	97.4 ± 0.8
SH-EP	Non-amplified	1.06 ± 0.01	85.6 ± 12.8
WAC2	Amplified	0.23 ± 0.01	36.1 ±7.2

 Next we evaluated the *in vitro* replication rates of M002 in the neuroblastoma cell lines. For multi-step viral recovery, monolayers of SK-N-AS or SK-N-BE(2) cells were infected with M002 at a MOI of 0.1 PFU/cell, and at 6, 24, 48, 72 hours post-infection, viral replication was determined. As shown in Figure 1. **B**, after 72 hours of infection, M002 replicated to a titer 2 logs higher in the SK-N-BE(2) cell line compared to time zero. Similar trends for M002 infectivity were seen with the SK-N-AS cell line (Figure 1. **B**). Single step viral recovery experiments were also performed. SK-N-AS and SK-N-BE(2) cell lines were both infected with MOI of 10 PFU/cell. By 24 hours post-infection with M002 there were significant viral titers in both cell lines (Figure 1. **C**).

**Figure 2:**
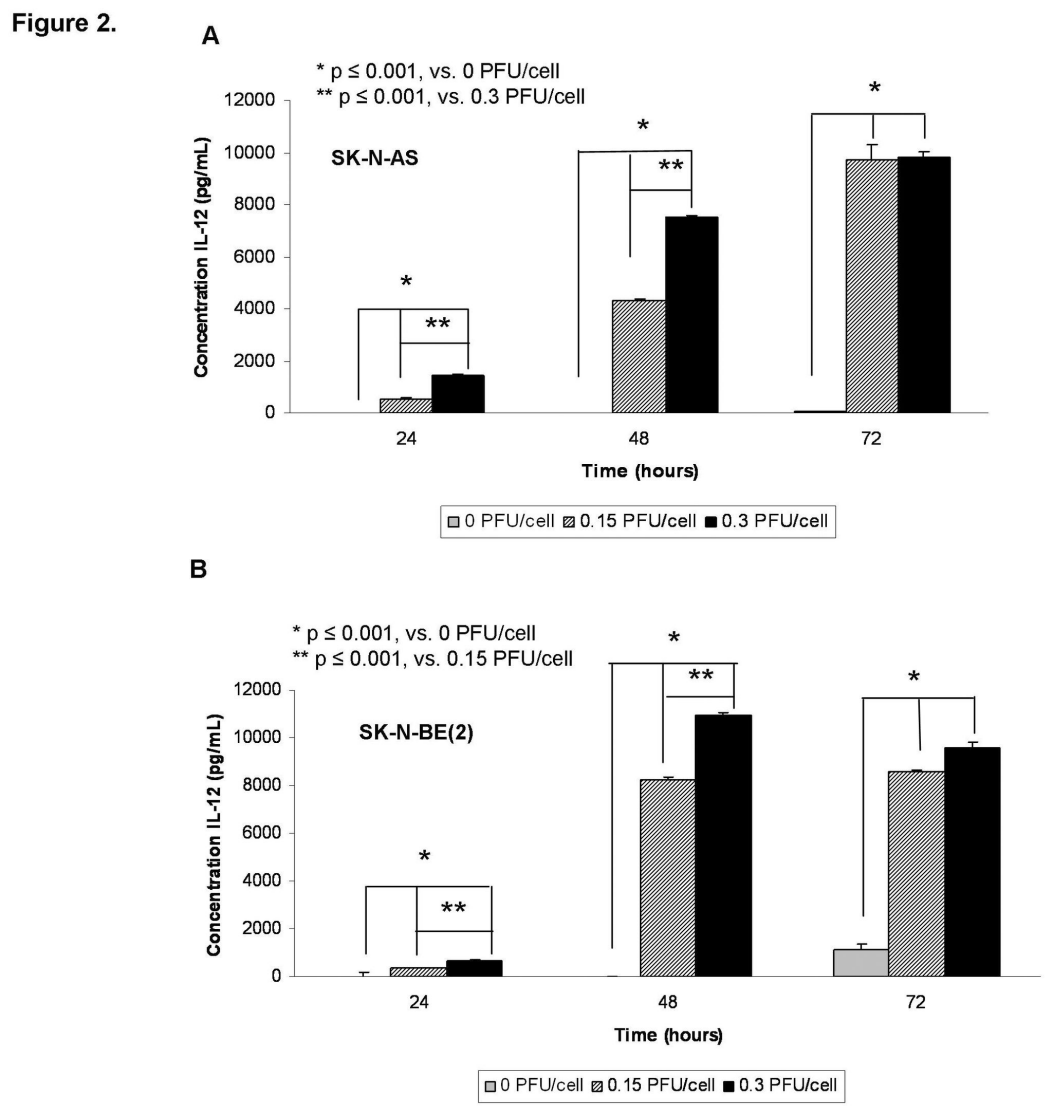
M002 infection resulted in murine IL-12 production in human neuroblastoma cell lines. Murine IL-12 production was determined in SK-N-AS **A** and SK-N-BE(2) B cell lines following treatment with M002 oHSV. Cell lines were infected with M002 at 0, 0.15 or 0.30 PFU/cell. At 24, 48, and 72 hours post-infection, the supernates were collected and concentrations of murine IL-12 determined by ELISA. Data reported as mean ± standard error of the mean. There was a significant increase in murine IL-12 production in both cell lines as early as 24 hours post-infection.

 M002 was genetically engineered to produce murine IL-12, so we sought to determine the extent to which the infected human neuroblastoma cell lines would produce the encoded foreign murine IL-12 protein, further verifying viral infection. The SK-N-AS and SK-N-BE(2) cells were infected with M002 at 0.15 or 0.3 PFU/cell. After 24, 48, and 72 hours of infection, the supernates were collected and a commercially available murine IL-12 ELISA kit was utilized to detect IL-12 production. M002 infection of both cell lines resulted in a significant increase in the production of murine IL-12 as early as 24 hours after infection (Figure 2. **A**, **B**). In the SK-N-AS cell line, infection with 0.15 PFU/cell for 24 hours resulted in a concentration of murine IL-12 of 532 ± 45 pg/mL, significantly greater than baseline concentration of 0 ± 4 pg/mL (p ≤ 0.001) (Figure 2. **A**). Similar findings were seen with the SK-N-BE(2) cell line; infection with 0.15 PFU/cell for 24 hours resulted in a concentration of murine IL-12 of 352 ± 17 pg/mL significantly greater than baseline concentration of 0 ± 14 pg/mL (p ≤ 0.001) (Figure 2. **B**). These trends continued at all time points. In addition, doubling the M002 concentrations resulted in a significant increase in the production of murine IL-12 at 24 and 48 hours in both cell lines compared to the lower concentration of the virus (Figure 2. **A**, **B**). 

### *In vivo* neuroblastoma tumor studies

**Figure 3:**
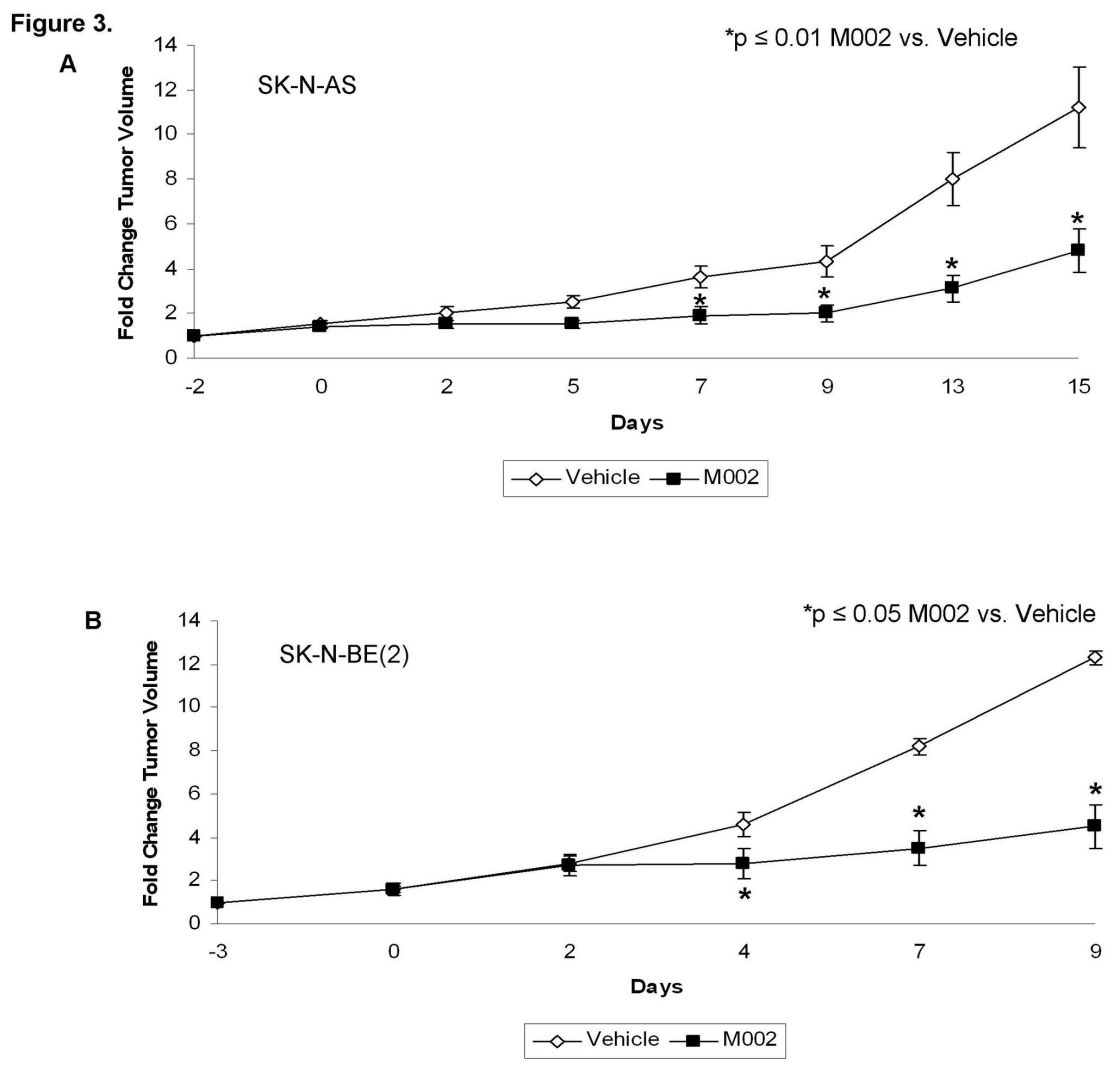
M002 treatment of neuroblastoma xenografts. **A** SK-N-AS or **B** SK-N-BE(2) human neuroblastoma tumor cells (2.5 ×10^6^ cells) in Matrigel™ were injected into the right flank of athymic nude mice. Once tumors reached 300 mm^3^, animals received an intra-tumoral injection of vehicle [PBS + 10% glycerol, 50μL (n=5)] or M002 virus [1 × 10^7^ PFU / 50μL (n=5)]. Tumor volumes were measured twice weekly [(width)^2^ × length]/2. Data reported as fold change in tumor volume ± standard error. In the SK-N-AS xenografts **A**, there was a significant decrease in tumor growth beginning at day 7 that continued to the end of the study. In the SK-N-BE(2) xenografts **B**, there was a significant decrease in tumor growth beginning at day 4 that continued to study completion.

To determine the *in vivo* effects of M002 treatment, a nude mouse model of SK-N-AS and SK-N-BE(2) neuroblastoma xenografts was utilized. Once xenografts reached 300 mm^3^, tumors were injected with vehicle (50 μL) or M002 (1 × 10^7^ PFU/50 μL). Tumor volumes were measured biweekly. Fold change in tumor volume was defined as the tumor volume at the specified time divided by the initial tumor volume. In the SK-N-AS xenografts, there was a significant decrease in tumor growth following M002 treatment, which became evident at 7 days post-treatment (Figure 3. **A**). In the SK-N-BE(2) xenografts, there was also a significant decrease in tumor growth that became significant at 4 days post treatment (Figure 3. **B**). 

 It has been demonstrated that M002 exerts its effects partially through the production of IL-12 and immune modulation [11]. Knowing that the immune response in nude mice would be limited to natural killer (NK) cells, we used flow cytometry to determine if there was a significant contribution from the immune system to our findings of decreased tumor growth in the M002 treated tumors. There was no significant difference in NK cell count in tumors treated with vehicle versus those treated with M002 (10.4 ± 5.8 % vs. 9.1 ± 3.7 %, N.S.); not an unexpected finding considering we used immunodeficient mice for these experiments. 

**Figure 4:**
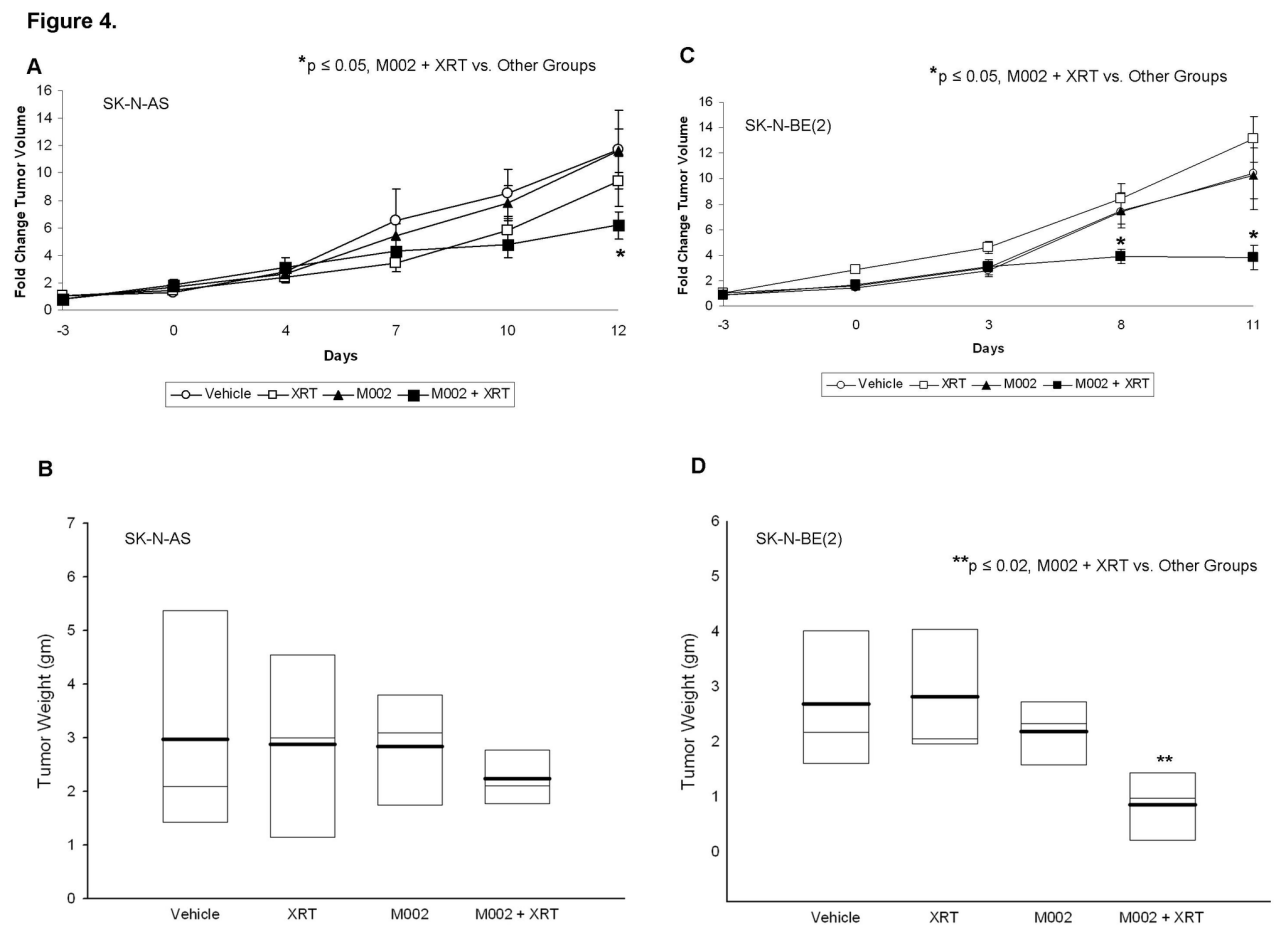
Combined M002 and irradiation to neuroblastoma xenografts. **A** SK-N-AS or **C** SK-N-BE(2) (2.5 ×10^6^ cells) in Matrigel™ were injected into the right flank of athymic nude mice. Once tumors reached 300 mm^3^, animals received an intra-tumoral injection of vehicle [PBS + 10% glycerol, 50μL (n=10)] or M002 virus [1 × 10^4^ PFU / 50μL (n=10)]. Half of each group was also treated with 3Gy external beam radiation at the time of M002 injection (XRT). Tumor volumes were measured twice weekly and reported as fold change in tumor volume ± standard error. There was a significant decrease in tumor volume in the animals treated with combined modalities for both tumor types (**A** and **C**). At the completion of the study, tumors were harvested and weighed. **B** In the SK-N-AS xenografts, the tumors with combined therapy tended to weigh less that those treated with vehicle or either modality alone (median = light bars, mean = dark bars), but did not reach significance statistically. **D** In the SK-N-BE(2) xenografts, the tumor weight was significantly less in the animals that received both modalities over those that were treated with vehicle or either therapy alone (median = light bars, mean = dark bars).

**Figure 5:**
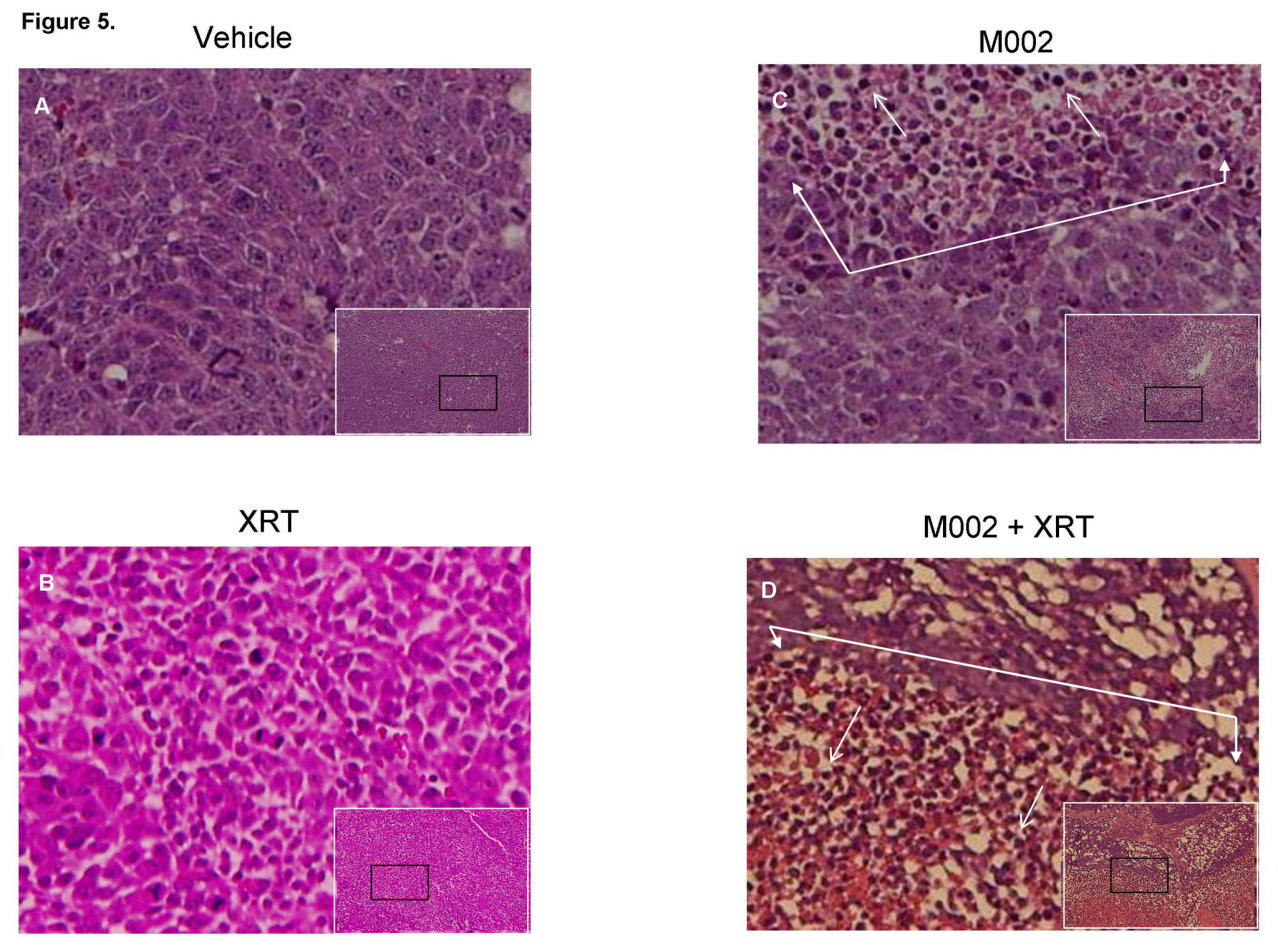
Histological examinations of combined M002 and irradiation in neuroblastoma xenografts. Small white boxes are representative photomicrographs (10 ×) of hematoxylin eosin stained SK-N-BE(2) neuroblastoma xenografts. The small black boxes are the areas of the tumor that are presented in the larger photomicrographs (40 ×). In the xenografts treated with vehicle **A** or XRT **B** alone, there were dense sheets of tumor cells with no necrosis, fibrosis or inflammatory cell infiltrate. **C** In the xenografts treated with M002 alone there was necrosis (area encompassed by closed white arrows) and inflammatory cell infiltrate (open white arrows). **D** In the tumors treated with M002 combined with XRT, there was more marked necrosis (area encompassed by closed white arrows) and more inflammatory cell infiltrate (open white arrows).

 The addition of low dose radiation has been shown to increase the activity of oHSV in malignant gliomas [15,20,21], thus we sought to determine if the addition of low dose radiation would enhance the efficacy of M002 in neuroblastoma xenografts. SK-N-AS and SK-N-BE(2) xenograft tumors were treated with a low dose of M002 (1 × 10^4^ PFU/50 μL) followed in 24 hours either with or without the addition of low dose irradiation (XRT). Three gray (3 Gy) was chosen as other investigations have shown that HSV replication increased in a dose dependent fashion following irradiation with 2 to 5 Gy, with no additional effects seen after 5 Gy [21]. In the SK-N-AS xenografts, treatment with low dose M002 with 3 Gy irradiation resulted in a significant decrease in tumor volume compared to xenografts treated with M002 (low dose) alone, vehicle with XRT, or vehicle alone (Figure 4. **A**). In addition, the tumor weights were decreased in these animals compared to the other groups, however, this decrease was not statistically significant (Figure 4. **B**). Similar findings were observed with the SK-N-BE(2) xenografts, but in these xenografts, there was a significant decrease in both tumor volume and in tumor weight in those animals that received M002 with XRT (Figure 3. C, D). Treatment with low dose M002 alone, vehicle with XRT, or vehicle alone did not alter the growth of these xenografts. Histological examination of the xenograft tumor specimens showed an increase in tumor necrosis and fibrosis (*closed arrows*), and inflammatory cell infiltrate (*open arrows*) in the xenografts treated with M002 (Figure 5. **C**) compared to those treated with vehicle or XRT alone (Figure 5. **A**, **B**, respectively). These histological changes were even more marked in the xenografts treated with the combination of M002 and XRT (Figure 5. **D**). 

**Figure 6:**
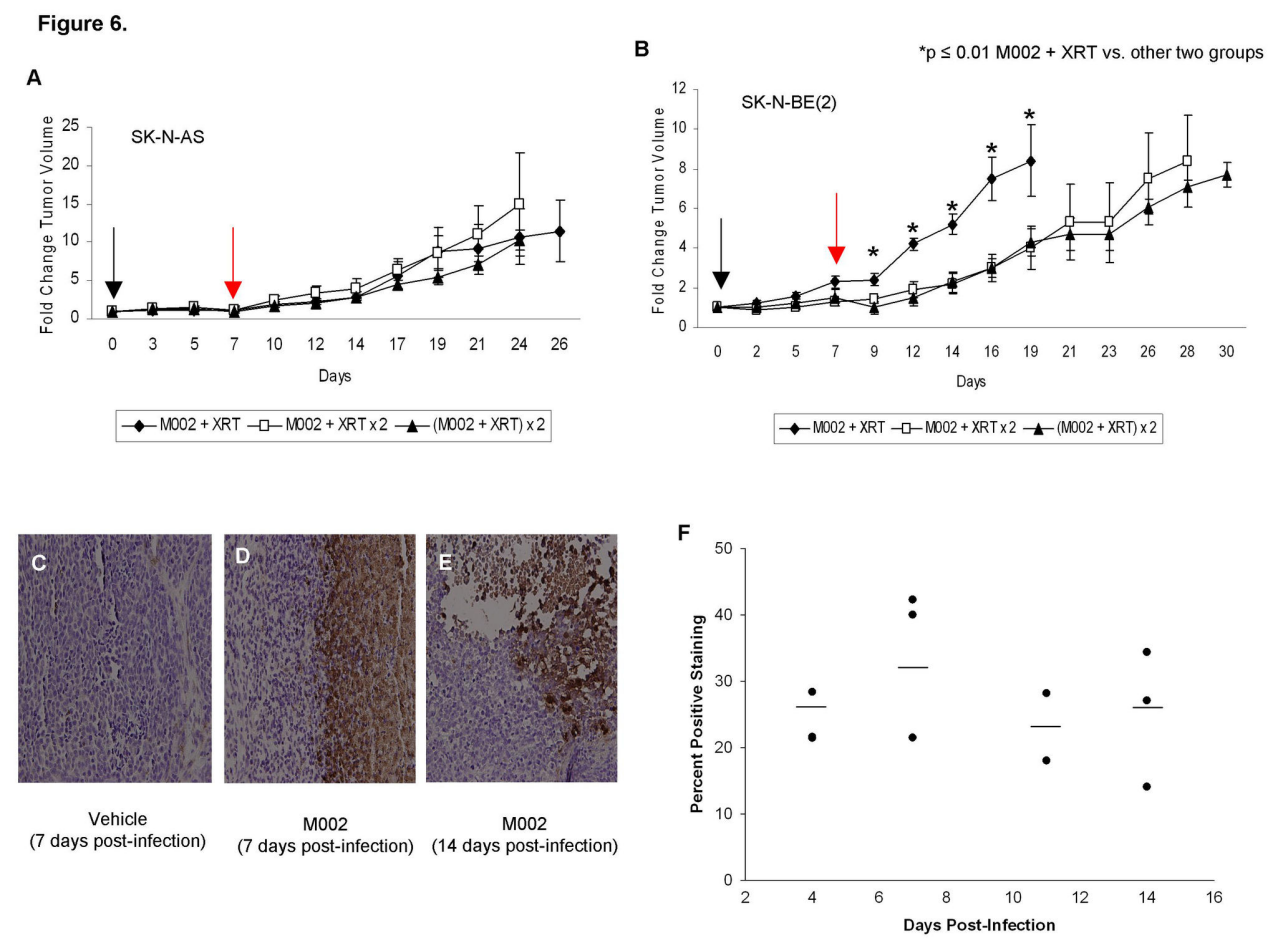
Repeat administration of M002 and irradiation to neuroblastoma xenografts. Established right flank xenografts of **A** SK-N-AS or **B** SK-N-BE(2) were treated with intra-tumoral injection of low dose M002 virus [1 × 10^4^ PFU / 50μL (N=15)] followed by low dose XRT (3 Gy) directed at the tumor (time 0, *black* *arrow*). One week later (red arrow), the animals were divided into 3 groups (n=5, each). One group received no further treatment (M002 + XRT). The second group received a repeat low dose of XRT (3 Gy) directed to the tumor (M002 + XRT × 2), and the third group received a second dose of intratumoral M002 (1 × 10^4^ PFU / 50μL) with a low dose XRT (3 Gy) [(M002 + XRT) × 2]. Tumor volumes were measured twice weekly [(width^2^ × length)/2]. Data reported as fold change in tumor volume ± standard error. **A** There was no difference in tumor growth in the SK-N-AS xenografts between the 3 treatment groups. **B** In the SK-N-BE(2) xenografts there was a significant decrease in tumor growth in the animals treated with both repeat low dose XRT (M002 + XRT × 2) or repeat M002 with low dose XRT [(M002 + XRT) × 2], but there was no difference between the animals that received repeat XRT and those that received an additional dose of M002. C, D, **E** Representative photomicrographs of SK-N-BE(2) xenografts immunostained for HSV-1 following 7 days treatment with vehicle or 7 and 14 days post-infection with M002. C No HSV-1 was detected in the vehicle treated xenografts. **D** In xenografts treated with M002 and XRT and harvested at 7 days post-infection, there was HSV-1 staining present (brown stain). **E** HSV-1 was still detected by immunohistochemistry (brown stain) in xenografts that were treated and harvested 14 days following M002 treatment. **F** Slides stained for HSV-1 were evaluated with ImageJ software and percent positive staining determined. There was a slight increase in the amount of positive staining at 7 days post-infection with M002 compared to the other time points, but this was not statistically different from any of the other time points. Importantly, there was no significant decrease in the amount of positive staining in those xenografts treated with repeat XRT and harvested at 11 or 14 days following original M002 injection compared to those harvested 7 days following M002 injection (34.6 ± 6.7% vs. 25.2 ± 6.0%, day 7 post-M002 vs. day 14 post-M002, p = 0.17).

 In a separate series of studies, SK-N-AS and SK-N-BE(2) xenografts were treated with either a repeat dose of 3Gy XRT alone or a repeat dose of M002 with 3Gy XRT one week after their initial treatment with M002 and 3Gy XRT. We found in both cell lines that a single repeat dose of irradiation 7 days after initial M002 injection was as effective at reducing tumor growth as repeating the dose of M002 (Figure 6. **A**, **B**). 

**Figure 7:**
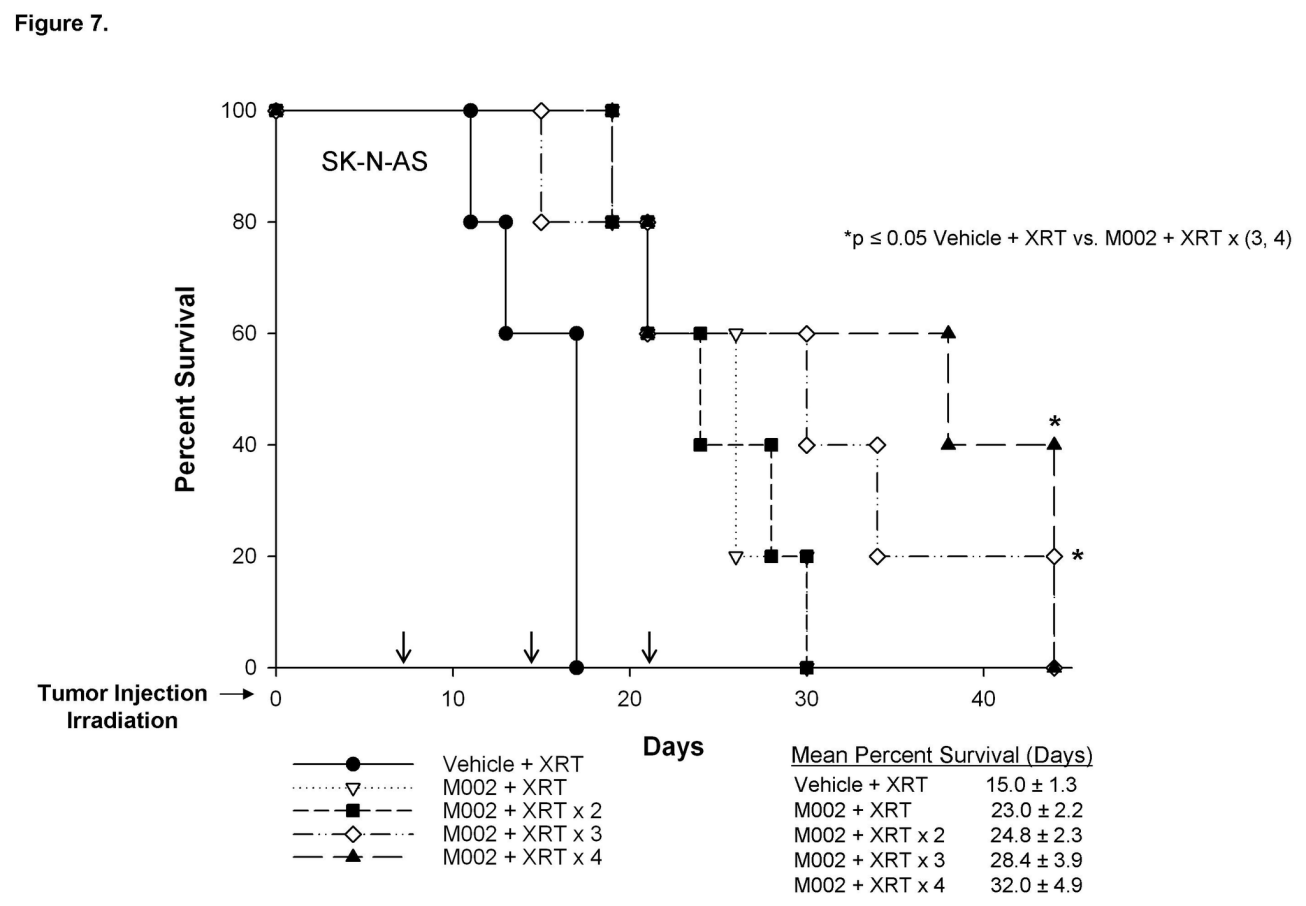
Survival of nude mice with SK-N-AS neuroblastoma xenografts treated with repeated XRT. Established SK-N-AS right flank xenografts (N=25) were treated with vehicle (50 μL) or M002 (1 × 10^4^ PFU/50 μL) and low dose ionizing radiation (XRT) (3 Gy) at Day 0. Subsequent low doses of XRT (3 Gy) were given at 7 day intervals (black arrows) to all vehicle treated animals (n=5); the M002 treated animals were divided into four groups (n=5 per group) to receive either no further treatment or an additional 1, 2, or 3 doses of low dose XRT at 7 day intervals (black arrows). The mean survival time in the M002 treated animals increased with each subsequent dose of XRT, but did not reach statistical significance. However, 40% (2/5) of the mice that received M002 XRT × 4 regimens and 20% (1/5) of those receiving M002 × 3 doses survived 44 days. Animals that were treated with M002 trended toward increased survival over those treated with irradiation alone, and this trend became statistically significant in the animals that received M002 with 3 and 4 doses of irradiation.

 Immunohistochemical staining for HSV-1 was performed on xenografts from animals euthanized 4 or 7 days following M002 injection, and after an additional 4 and 7 days following the repeated dose of irradiation (11 and 14 days following M002 injection, respectively). Slides were evaluated with ImageJ software and percent positive staining determined. There was a slight increase in the amount of positive staining at 7 days post-infection, but this was not statistically different from any of the other time points. Importantly, there was no significant decrease in the amount of positive staining in those xenografts treated with repeat XRT and harvested at 11 or 14 days following original M002 injection compared to those harvested 7 days following M002 injection (34.6 ± 6.7% vs. 25.2 ± 6.0%, day 7 post-M002 vs. day 14 post-M002, p = 0.17) (Figure 6. C, D, **E**, **F**). Therefore, we hypothesized that repeat small doses of irradiation (3 Gy) would result in a therapeutic response. To test this hypothesis, flank xenografts of SK-N-AS or SK-N-BE(2) cells were established in nude mice and once tumors reached 300 mm^3^, the animals received intratumoral injection of vehicle or M002 (1 × 10^4^ PFU/50 μL) followed by 3 Gy of irradiation to the tumor. Beginning one week after initial treatment, all of the vehicle treated animals received a repeat low dose of ionizing radiation (3 Gy) to the tumor once per week for the next 3 weeks. The M002 treated animals were randomized (n=5 per group) to receive either no further treatment, or an additional 1, 2, or 3 exposures to 3 Gy XRT at weekly intervals. Tumor volumes were measured twice weekly and animals were euthanized when tumor volumes reached 1500 mm^3^, the size allowed by IACUC protocol. In the mice with SK-N-AS xenografts treated with M002, mean survival times increased with each subsequent dose of irradiation, although the increase in median survival at 44 days in the mice that received M002 and 4 doses of XRT was not statistically different compared to animals that received M002 and 1 dose of XRT (Figure 7.). However, 40% (2/5) of the mice that received the M002 XRT × 4 regimen and 20% (1/5) of those receiving M002 XRT × 3 doses survived to 44 days. Also, survival was increased in the animals that were treated with M002 and 3 or 4 subsequent doses of XRT when compared to those treated with vehicle and XRT (Figure 7.). There was a significant decrease in the fold change in tumor volume in the animals treated in the M002 XRT × 1 or M002 XRT × 2 groups when compared to vehicle with XRT (Figure S1. **A**), despite the lack of survival difference, again demonstrating a significant impact of M002 on tumor growth. There was also a significant decrease in tumor volume in the animals that received M002 XRT × 4 versus those that received only 1 or 2 doses of XRT with their M002 (Figure S1. **A**). 

**Figure 8:**
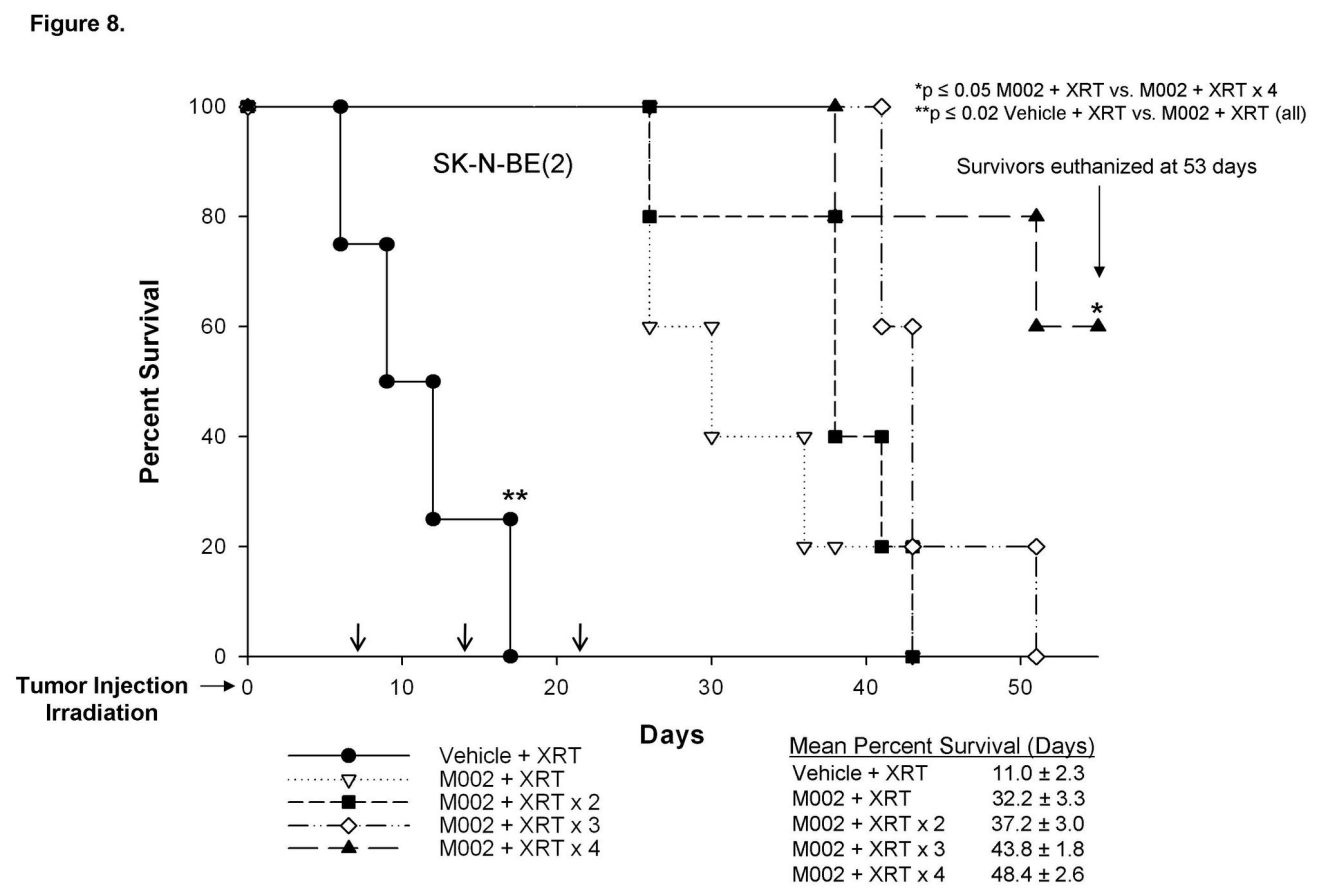
Survival of nude mice with SK-N-BE(2) neuroblastoma xenografts treated with M002 and repeated XRT. Established SK-N-BE(2) right flank xenografts (N=25) were treated with vehicle (50 μL) or M002 (1 × 10^4^ PFU/50 μL) and low dose ionizing radiation (XRT) (3 Gy) at Day 0. Subsequent low doses of XRT (3 Gy) were given at 7 day intervals (black arrows) to all vehicle treated animals (n=5); the M002 treated animals were divided into four groups (n=5 per group) to receive either no further treatment or an additional 1, 2, or 3 doses of low dose XRT at 7 day intervals (black arrows). Three surviving animals, all in the M002 XRT × 4 regimen were euthanized at 53 days, with small mean tumor volume (450 ± 131.1 mm^3^). The mean survival time increased with each subsequent dose of XRT, reaching statistical significance after four doses. M002 with XRT at all groups showed a survival advantage over vehicle with subsequent doses of XRT.

 In the SK-N-BE(2) tumors, mean survivals were also increased with each subsequent repeat dose of irradiation (Figure 8.). In the SK-N-BE(2) xenografts, there was a significant survival advantage in the animals that were treated with M002 and XRT at all XRT doses when compared to those treated with vehicle and XRT. Additionally, there was a survival advantage for animals treated with M002 and 4 doses of XRT compared to those receiving M002 and only a single dose of XRT (32.2 ± 3.3 vs. 48.4 ± 2.6 days, M002 XRT vs. M002 XRT × 4, p ≤ 0.05). Three survivors in the M002 × 4 group were euthanized at 53 days, the time that the last animal in the M002 XRT × 3 group was euthanized. At the time of sacrifice, the mean tumor volumes for the M002 XRT × 4 animals were small at 450.0 ± 131.1 mm^3^. When examining change in tumor volume, there was a significant decrease in those animals that received M002 and XRT at all doses when compared to those that were treated with vehicle and repeated doses of XRT (Figure S1. **B**). The animals that were treated in the M002 XRT × 4 also had significantly less increase in tumor volumes compared to all of the other M002 XRT groups (Figure S1. **B**). 

**Figure 9:**
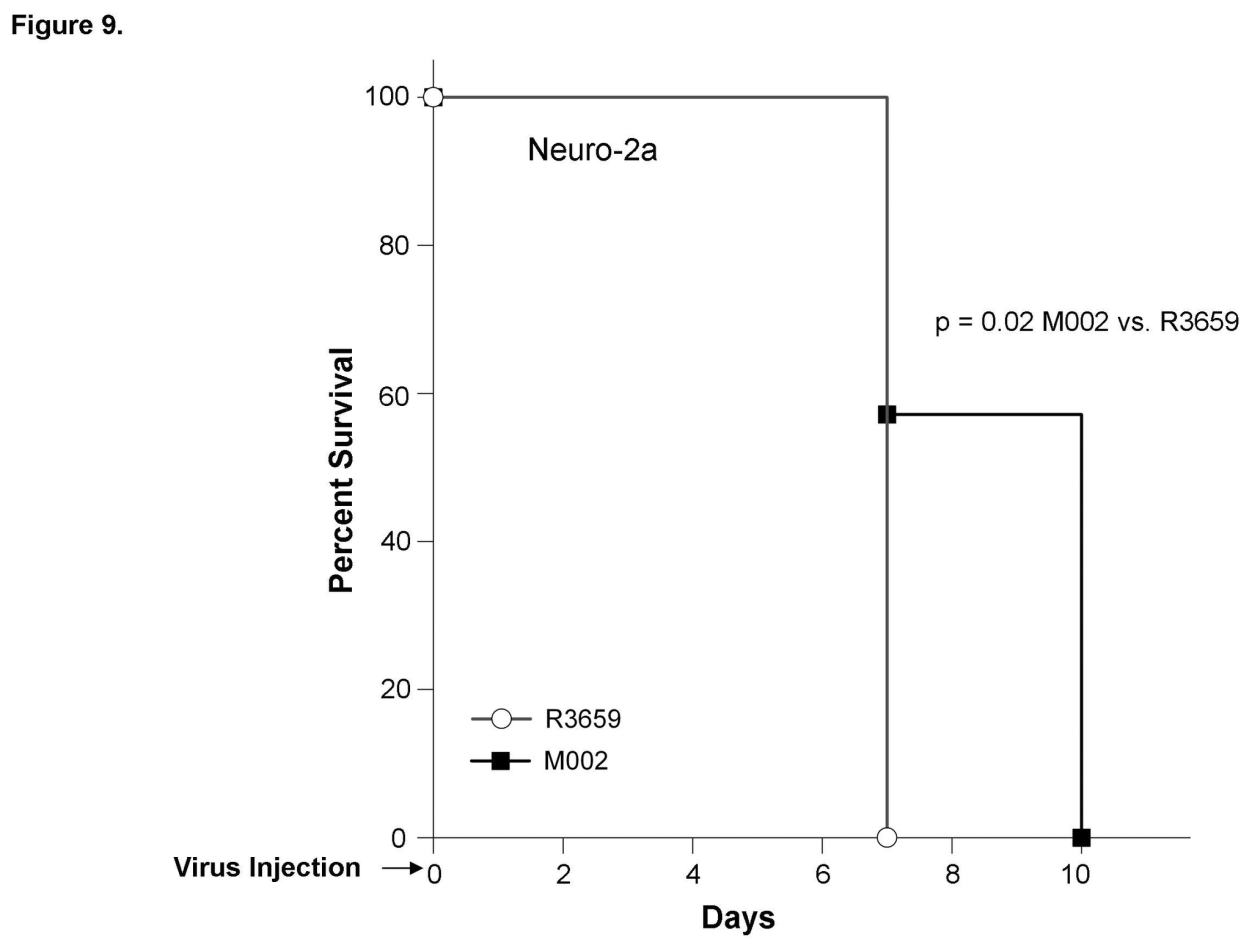
Survival of immunocompetent AJ mice with Neuro-2a murine neuroblastoma tumors treated with R3659 or M002. Neuro-2a murine neuroblastoma tumor cells (5 × 10^5^ cells) were injected into the right flank of immunocompetent syngeneic AJ mice. Once tumors reached 100 mm^3^, animals received an intra-tumoral injection of R3659 [parent virus, 1 × 10^7^ PFU / 50μL (n=8)] or M002 [IL-12 engineered virus, 1 × 10^7^ PFU / 50μL (n=7)] and animals were followed for survival. The median survival of the animals with tumors treated with R3659 was 7 days and was significantly decreased from the median survival of animals with M002-treated tumors (10 days, p = 0.02).

 Since the M002 oHSV has the gene for murine IL-12 inserted, we wished to determine if the addition of IL-12 had any effect upon neuroblastoma tumorigenicity *in vivo* when compared to the parent virus, R3659. An immunocompetent syngeneic model of murine neuroblastoma was required to accomplish this aim. Neuro-2a murine neuroblastoma tumors were established in the subcutaneous space of the right flank of syngeneic AJ mice. Following virus injection, the median survival of animals treated with the parent R3659 virus was 7 days. In contrast, mice with M002-injected tumors had a median survival of 10 days (p = 0.02) (Figure 9.). 

### CD111 expression and phosphorylation of p38 and STAT1

**Figure 10:**
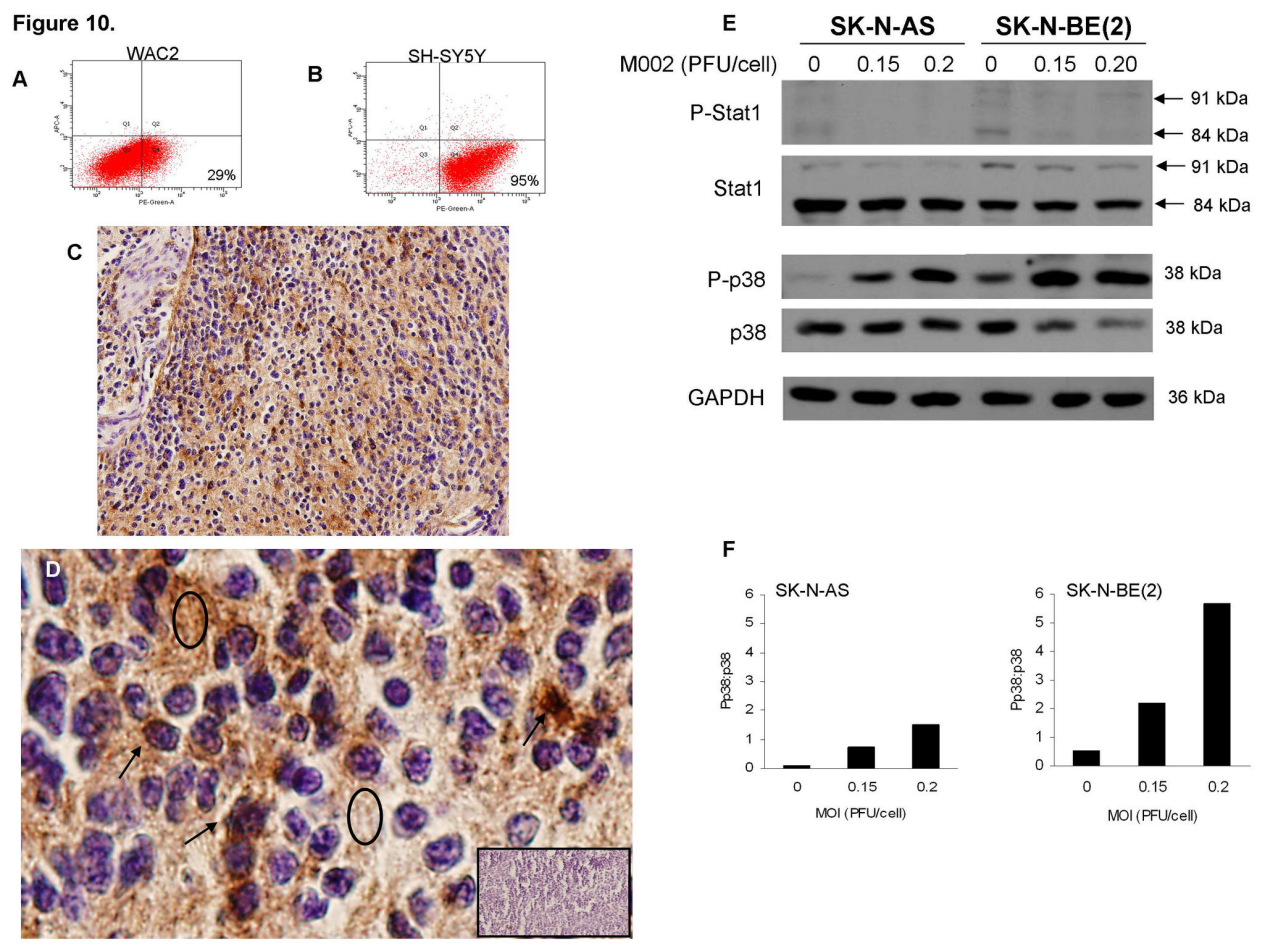
CD111 expression and phosphorylation of p38 and STAT1. Neuroblastoma cells were stained for CD111 expression and evaluated with FACS. Two representative scatterplots for WAC2 **A** and SH-SY5Y **B** neuroblastoma cell lines. CD111 (lower right quadrant) was detected in all cell lines tested. **C** Immunohistochemistry was performed on formalin-fixed, paraffin-embedded human neuroblastoma specimens. Positive staining (brown) in a representative photomicrograph (10 ×). **D** A magnification (40 ×) of **C** revealed that CD111 staining was present in the cells (black arrows) and not just limited to the surrounding neuropil (black ovals). Negative controls were included with each stain series (inserted black rectangle). **E** SK-N-AS and SK-N-BE(2) cells were treated with or without M002 and immunoblotting for total and phosphorylated Stat1 and p38 was performed. Phosphorylation of Stat1 was not increased following M002 treatment in either cell line. Phosphorylation of p38 was increased in both cell lines after M002 treatment. F Densitometry was used to further show the differences in p38 phosphorylation following M002 treatment. Bands were normalized to those of GAPDH and represented as the density of the phosphorylated p38 band compared to that of the total p38 band. With increasing MOI, phosphorylation of p38 increased in both cell lines, and this increase was more marked in the SK-N-BE(2) cell line.

CD111 [poliovirus receptor-related protein 1 (PRR1, nectin-1)] is the primary human herpes virus entry mediator used by HSV-1 for entry into the cell [22]. To determine whether a difference in CD111 expression may have impacted the response of the tumor cell lines to M002, FACS analysis was used to determine the CD111 expression of several neuroblastoma cell lines, both *MYCN* amplified and non-amplified. Representative FACS scatterplots for two neuroblastoma cell lines, WAC2 (amplified *MYCN*) and SH-SY5Y (non-amplified *MYCN*) is presented in Figure 10. **A**, **B**, respectively. CD111 was detected in all five neuroblastoma cell lines tested and ranged from 9.8 to 97.4% expression (Table 1). Since the CD111 expression in the SK-N-AS cell line was low, we investigated CD112 (poliovirus receptor-related 2, nectin-2) expression, another receptor that the oHSV may utilize for cell entry [22]. The expression of CD112 in the SK-N-AS cell line by FACS analysis was 75 ± 0.4%.

 To provide the rationale for clinical application and relevance of oHSV therapy for human neuroblastoma, we performed immunohistochemistry for CD111 on 18 human neuroblastoma samples. The CD111 staining was scored by a pathologist blinded to the specimens and the mean stain scores were compiled based upon stain intensity. Over 78% of the specimens had positive staining for CD111. The distribution of staining based upon known biologic risk factors for neuroblastoma was presented in Table 2. Neuroblastoma tumors that are of advanced Stage [23,24], *MYCN* amplified [25,26] or that are diagnosed after 18 months of age [27] carry the worst prognosis. Since aggressive, metastatic disease will most likely be the initial target of oHSV therapy, we compared stain scores between known poor prognostic variables. There were no statistical differences in stain scores between tumors that were advanced stage (INSS Stage 4), *MYCN* amplified, or in children greater than 18 months of age at diagnosis (Table 2). Staining was not significantly different in the tumors that had undergone previous chemotherapy compared to those that had not (data not shown). A representative photomicrograph of the CD111 staining was presented at 10× (Figure 10. **C**). Magnification to 40× showed staining was present in the cells themselves (*black arrows*) and not limited to the surrounding neuropil (*black ovals*) (Figure 10. **D**). A negative control was included for each staining run (Figure 10. **D**, *rectangular insert lower right corner*). These data clearly demonstrated that the majority of human neuroblastoma cell lines and human tumors tested expressed the HSV entry receptor, CD111. 

**Table 2:** Immunohistochemical staining CD111^+^ by neuroblastoma biologic risk factors.

**Biologic Risk Factors**	**Immunostaining**	**Stain Score**	**p-value**
	**Cohort (N=18)**	**(Mean ± SEM)**	
INSS Stage			
1, 2, 3, 4S	10	2.2 ± 0.4	0.78
4	8	2.0 ± 0.6	
*MYCN* Amplified			
No	7	2.4 ± 0.3	0.18
Yes	7	1.4 ± 0.6	
(Unknown)	(4)		
Age			
< 18 months	11	2.2 ± 0.4	0.15
≥ 18 months	7	1.3 ± 0.5	

 Previous investigators have shown that upregulation of STAT1 was associated with decreased response to oHSV [9]. Other investigators have shown that decreased phosphorylation of p38 was also associated with resistance to oHSV [9,20]. Therefore, to determine if these proteins played a potential role in the difference in responses of the cell lines to oHSV, immunoblotting was utilized to determine their expression and phosphorylation. There was no increase in STAT1 activation following M002 treatment in either cell line (Figure 10. **E**, **F**). Additionally, neither cell line demonstrated a decrease in the phosphorylation of p38 after infection with M002 (Figure 10. **E**, **F**). In fact, p38 phosphorylation increased with M002 treatment in both cell lines, and densitometry showed it was more increased in the SK-N-BE(2) cell line than in the SK-N-AS cell line (Figure 10. **F**). Therefore, STAT1 and p38 did not appear to be potential mechanisms of cellular resistance to oHSV in these neuroblastoma cell lines. 

## Discussion

Despite therapeutic advances, long term survival has not significantly improved over the last 20 years for children with advanced stage or metastatic neuroblastoma. Clearly, novel therapies are needed for this devastating tumor, and viral and immune therapies are among the alternatives currently being investigated [7,28]. Multiple viruses have been proposed in the treatment of neuroblastoma and have shown various levels of efficacy [29]. Toyoda and colleagues utilized a live-attenuated poliovirus and showed that this virus killed 27 of 29 neuroblastoma cell lines and significantly reduced the growth of neuroblastoma xenografts in mice [4]. Gil reported decreased growth in murine neuroblastomas that were treated with oncolytic vaccinia virus in combination with photodynamic therapy [5]. Neither of these viruses has advanced to human trials for neuroblastoma. Concerns over the possible lack of efficacy with poliovirus due to childhood vaccination and difficulties with endothelial transport due to the size of the vaccinia viral particles have been some of the issues raised. Preclinical and preliminary clinical studies have been conducted using adenovirus as treatment for neuroblastoma [6,30]. Although adenovirus therapy for neuroblastoma has shown limited success, systemic therapy is questionable since the virus does not localize to tumor tissue without a vector for viral delivery. Because of the known pitfalls associated with other oncolytic viruses, we have chosen to study an oncolytic herpes simplex virus for neuroblastoma. Parikh and colleagues previously published their data showing that neuroblastoma models were more susceptible to a mutant oHSV than to adenovirus [7], further supporting our investigations with oHSV in neuroblastoma. 

 Previous studies have shown that oncolytic herpes simplex virus infected both murine and human neuroblastoma cells [9,10]. The murine neuroblastoma cell line, Neuro2a, has been used by a number of investigators to study oHSV effects in immunocompetent mouse models [31-33]. Chung et al saw successful infection of a human neuroblastoma cell line, SK-N-SH, with a gamma 34.5 mutant HSV-1 [34]. In our study, we expanded the number of cell lines studied and demonstrated that these human neuroblastoma cell lines were sensitive to oncolysis by a novel, replication competent oHSV, M002. We also demonstrated that M002 was able to infect these neuroblastoma cell lines at low MOI. 

 Although virotherapy alone may be a viable therapeutic option for neuroblastoma, modifications, such as the addition of exogenous cytokines, could further contribute to the anti-tumor effects. The oHSV utilized in these studies was encoded with the gene for murine IL-12, and the levels of murine IL-12 that were produced by both cell lines were over several nanograms per milliliter. Interleukin-12 (IL-12) is best known as a T-lymphocyte and NK cell activator but also has anti-tumor effects on neuroblastoma. Siapati et al. transfected murine neuroblastoma cells to express IL-12 and significantly decreased their *in vivo* tumorigenicity [35]. In addition, Redlinger reported that in mice with established tumors, inoculation with an IL-12-secreting neuroblastoma cell line resulted in an anti-tumor response in the animals [36]. In the current studies, despite high levels of murine IL-12 production following cell infection *in vitro*, we were not able to document a significant increase in natural killer (NK) cell infiltration into the human tumors in the *in vivo* setting using the nude mouse model. We attributed these findings to the fact that we were utilizing an immunodeficient mouse model for our studies of human tumor cell lines. When an immunocompetent murine model was utilized in the study of Neuro-2a murine neuroblastoma, it was clear that the addition of IL-12 provided a significant survival advantage over the use of the parent virus (Figure 9.), presumably by promoting an inflammatory response. These findings clearly provide novel and new results in the study of oHSV in neuroblastoma. Finally, in other studies in our laboratory with immunocompetent mice and another syngeneic murine neuroblastoma cell line (NXS2), we were able to show a significant reduction in tumor growth (Figure S1. **C**) and a robust NK response when tumors were treated with M002 (7.7 ± 1.5% vs. 29.6 ± 0.4%, NK positive cells, vehicle vs. M002, p ≤ 0.001). Further investigations are planned to characterize the contribution of the immune system to neuroblastoma cell killing with M002.

 An important, novel aspect of the current study was the investigation of CD111 (nectin-1) in human neuroblastoma cell lines and human tumor tissues. We detected CD111 by FACS in all 5 neuroblastoma cell lines that we tested and by immunohistochemistry in the majority of human specimens studied. There did appear to be a relation between CD111 detection and cell killing as depicted in Table 1. Previous work by colleagues in our group showed that CD111 was a critical entry molecule for oHSV in tumor cells, but not all tumors of the same histological type contained sufficiently high levels of CD111-positive cells to be infected such that oHSVs could produce an anti-tumor effect. In their study, it appeared that there was a breakpoint around 20% CD111-positive cells that determined whether or not there was a significant oncolytic effect on the tumor [37]. However, in the current study, despite low levels of CD111 in the SK-N-AS cell line, M002 was still very effective at cell infection and killing (Figure 1.) in that cell line. The obvious efficacy of M002 in the SK-N-AS cell line despite low CD111, suggested the potential for other receptors. Since it has been reported in the literature that certain laboratory strains of HSV-1 can use nectin-2 for cell entry [22], we chose to study CD112 expression in these cells, and found that it was highly expressed in the SK-N-AS cell line. 

 Until our study, there has been only a single report addressing the expression of nectin-1 in human neuroblastoma cell lines. Krummenacher and colleagues in 2000 reported that nectin-1 was present on the cell surface of two neuroblastoma cell lines, IMR5 and SY5Y, as documented by FACS analysis [38]. Also, little data exists in the literature addressing the expression of CD111 in human neuroblastoma tumor specimens, and to our knowledge, this study may be one of the first to document this finding. Documenting CD111 in human neuroblastoma specimens was an important step in demonstrating that the virus formulated for human use, M032, may be an appropriate therapeutic for children with neuroblastoma. 

 The oncogene, *MYCN*, is the most significant negative prognostic factor for neuroblastoma survival [25,26], and neuroblastoma tumors with *MYCN* amplification are more likely to be metastatic and to recur. This oncogene is amplified in about 30% of neuroblastoma tumors [39]. Since *MYCN* is a transcription factor, we considered whether or not *MYCN* could affect the expression of CD111 in neuroblastoma that would limit the clinical usefulness of M002. However, we did not find a correlation between amplification of the *MYCN* oncogene and the expression of CD111 protein in either the neuroblastoma cell lines or the primary human tissue specimens examined and CD111 expression was detected in both *MYCN* amplified and nonamplified tumors. This finding has significant clinical relevance as it implies that the virus should be effective in most neuroblastomas irrespective of *MYCN* status.

 Our studies showed that the combination of ionizing radiation with the administration of M002 enhanced the effects of M002 in neuroblastoma xenografts. Previous authors have noted that radiotherapy acts synergistically with other oncolytic HSVs [13,40-43]. For example, Chung and colleagues reported that combining ionizing radiation with R7020 recombinant HSV caused a greater reduction in hepatoma xenografts than either radiation or virus alone [16]. Andusumulli and colleagues showed that oHSV NV1066 when combined with 5 Gy external beam radiation significantly reduced H1299 lung xenograft tumors when compared to either modality alone [42]. In translating this therapy to the clinical setting, our findings are important as they show that combining both modalities may allow for significant dose reduction in both therapies that may decrease therapy related toxicities. 

 Another clinically relevant finding from our study was the ability to continue to limit tumor growth simply by administering repeated low doses of irradiation without the need for reinjection of the virus. Although our studies showed variable differences in the survival of some the animals following repeated doses of irradiation (Figures 7., 8.), when change in tumor volume was examined, there was a significant difference seen in the animals that received 3 additional doses of irradiation (Figure S1. **A**, **B**). These results were consistent with previous studies that demonstrated enhanced viral replication following irradiation led to greater tumor regression [16,21]. Therefore, in the clinical setting, repeated injections of virus may be obviated by the addition of repeated low dose irradiation, making the use of oHSV treatment for disease located in sites that are not readily amenable to repeated injections, such as intrathoracic or intraabdominal tumors, more feasible. The ability to continue to have viral activity through simple repeated small doses of irradiation provides a novel impact on the treatment of children with relapsed or recurrent neuroblastoma. 

 We attempted to delineate the mechanisms responsible for the differing responses of the two neuroblastoma cell xenografts to M002. One explanation may be related to the expression of CD111, the virus entry molecule. The percentage of SK-N-AS cells expressing CD111 was lower than that of the SK-N-BE(2) cells, but the calculated LD_50_ for M002 was similar in the two cell lines. In fact, the two cell lines had similar responses to M002 in the *in vitro* studies and in the *in vivo* study without irradiation. Most of the differences were noted in the *in vivo* studies where low dose M002 and irradiation was utilized, where the SK-N-BE(2) xenografts had a more marked response to the M002 treatment than the SK-N-AS xenografts. Therefore, there may be another explanation. Mezhir and colleagues reported that an intact p38 pathway was essential for the upregulation of late virus genes that followed radiation treatment combined with attenuated herpes simplex viruses [20]. We noted that the p38 pathway was activated in both cell lines following treatment with M002 (Figure 9. **E**), but this activation was more marked in the SK-N-BE(2) cell line compared to the SK-N-AS cells (Figure 9. **F**). The differences in p38 phosphorylation could explain the different responses of the xenografts to M002 treatment when combined with irradiation. This finding will certainly be the subject of future investigations. 

 In summary, we have shown that the oHSV, M002, can infect and replicate in human neuroblastoma cell lines. In addition, the entry mechanism for the virus was present not only in human neuroblastoma cell lines, but also in human tumor specimens. The response seen in murine neuroblastoma xenografts suggests that an oHSV with the addition of IL-12 may have potential for use in children with unresponsive or relapsed neuroblastoma. 

## Supporting Information

Figure S1 **M002 oHSV treatment of human neuroblastoma xenografts with and without ionizing radiation (XRT).** A SK-N-AS or B SK-N-BE(2) (2.5 × 10^6^) cells in Matrigel™ were injected into the right flank of athymic nude mice (N=25 per tumor type). Once tumors reached 300 mm^3^ (day 0), animals received an intra-tumoral injection of vehicle (50μL) (n=5) or M002 virus [1 × 10^4^ PFU / 50μL (n=20)] and low dose XRT (3 Gy) directed to the tumor. Each week subsequent low dose XRT (3 Gy) was administered to the vehicle treated tumors (black arrows). M002 treated tumors were divided into 4 groups (n=5 per group) to receive either no further treatment or an additional 1, 2, or 3 doses of XRT (black arrows). Tumor volumes were measured twice weekly and fold change in tumor volume was calculated based upon the size of the tumor at time 0. In the SK-N-AS xenografts **A**, there was a significant decrease in tumor volume in the animals that received M002 and 4 doses of XRT versus those that received only one or two doses of XRT with their M002. Animals that received vehicle and XRT had significantly larger tumor volumes than those treated with M002 and repeated XRT. B In the SK-N-BE(2) xenografts, tumor growth was significantly decreased in the animals given M002 and 4 doses of XRT compared to those that had been exposed to M002 with 1, 2, or 3 low doses of XRT (3 Gy). Animals treated with vehicle and XRT had significantly increased tumor volumes compare to all animals treated with M002 and any amount of XRT. C NXS2 murine neuroblastoma tumor cells (7.5 × 10^5^) were injected into the right flank of syngeneic AJ mice (N=10). Once tumors reached 300 mm^3^ (day 0), animals received an intra-tumoral injection of vehicle (50μL) (n=5) or M002 virus [1 × 10^7^ PFU / 50μL (n=5)]. Tumor volumes were measured twice weekly with calipers. There was a significant decrease in fold change in tumor volume in animals treated with M002 compared to vehicle.
(TIF)
