# Supplementary figures and images for: Preclinical Evaluation of Engineered Oncolytic Herpes Simplex Virus for the Treatment of Neuroblastoma
Source: PLoS One. 2013 Oct 10;8(10):e77753. doi: 10.1371/journal.pone.0077753 (PMC3795073; doi:10.1371/journal.pone.0077753)

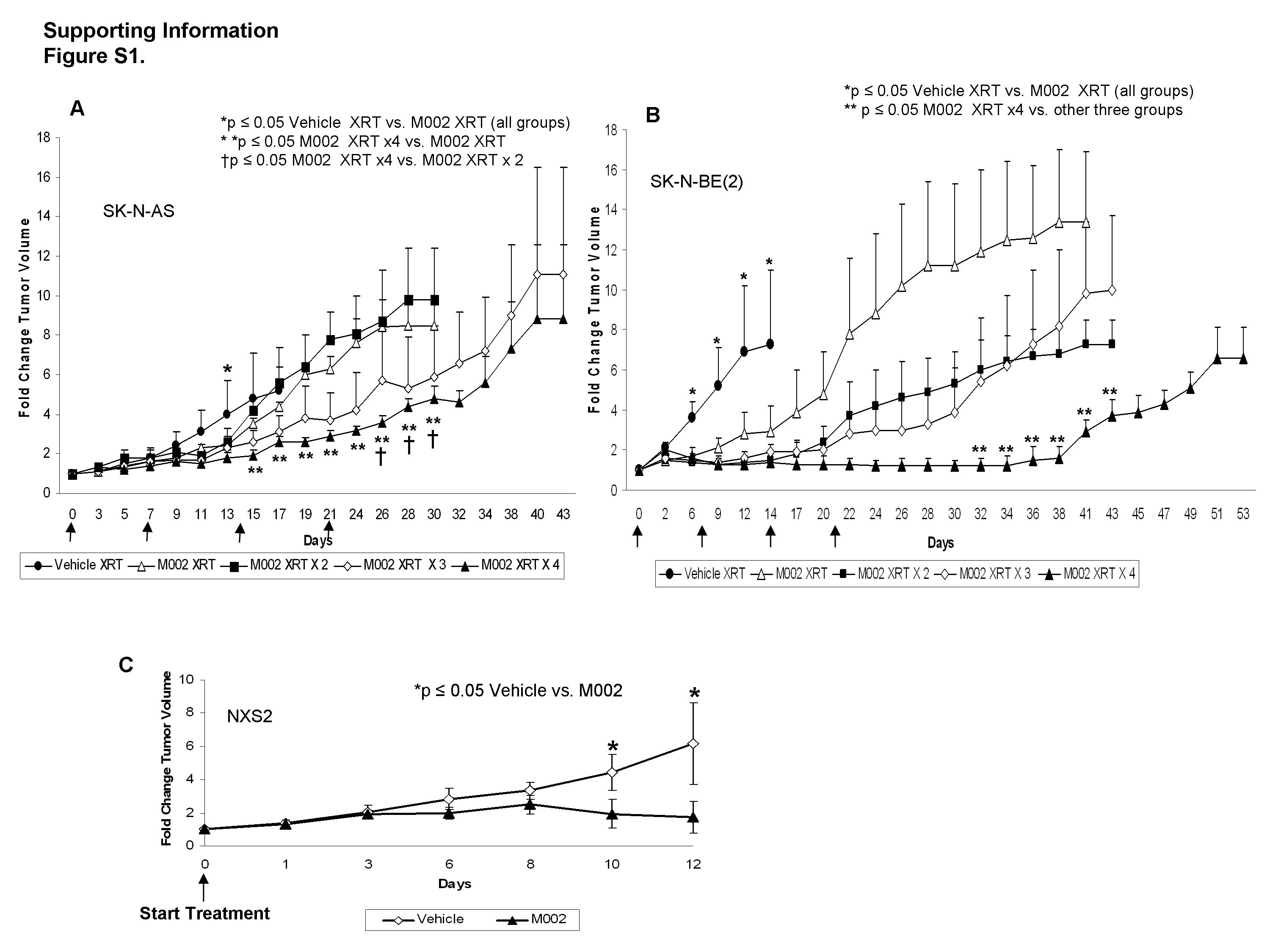

Supplement: Figure S1 — M002 oHSV treatment of human neuroblastoma xenografts with and without ionizing radiation (XRT). A SK-N-AS or B SK-N-BE(2) (2.5 × 10^6^) cells in Matrigel™ were injected into the right flank of athymic nude mice (N=25 per tumor type). Once tumors reached 300 mm3 (day 0), animals received an intra-tumoral injection of vehicle (50μL) (n=5) or M002 virus [1 × 10^4^ PFU / 50μL (n=20)] and low dose XRT (3 Gy) directed to the tumor. Each week subsequent low dose XRT (3 Gy) was administered to the vehicle treated tumors (black arrows). M002 treated tumors were divided into 4 groups (n=5 per group) to receive either no further treatment or an additional 1, 2, or 3 doses of XRT (black arrows). Tumor volumes were measured twice weekly and fold change in tumor volume was calculated based upon the size of the tumor at time 0. In the SK-N-AS xenografts A, there was a significant decrease in tumor volume in the animals that received M002 and 4 doses of XRT versus those that received only one or two doses of XRT with their M002. Animals that received vehicle and XRT had significantly larger tumor volumes than those treated with M002 and repeated XRT. B In the SK-N-BE(2) xenografts, tumor growth was significantly decreased in the animals given M002 and 4 doses of XRT compared to those that had been exposed to M002 with 1, 2, or 3 low doses of XRT (3 Gy). Animals treated with vehicle and XRT had significantly increased tumor volumes compare to all animals treated with M002 and any amount of XRT. C NXS2 murine neuroblastoma tumor cells (7.5 × 10^5^) were injected into the right flank of syngeneic AJ mice (N=10). Once tumors reached 300 mm3 (day 0), animals received an intra-tumoral injection of vehicle (50μL) (n=5) or M002 virus [1 × 10^7^ PFU / 50μL (n=5)]. Tumor volumes were measured twice weekly with calipers. There was a significant decrease in fold change in tumor volume in animals treated with M002 compared to vehicle. (TIF) [file pone.0077753.s001.tif]
